# Factors Associated With the Accuracy of Large Language Models in Basic Medical Science Examinations: Cross-Sectional Study

**DOI:** 10.2196/58898

**Published:** 2025-01-13

**Authors:** Naritsaret Kaewboonlert, Jiraphon Poontananggul, Natthipong Pongsuwan, Gun Bhakdisongkhram

**Affiliations:** 1 Institute of Medicine, Suranaree University of Technology, 111 University Avenue, Nakhon Ratchasima, 30000, Thailand, 66 44223956

**Keywords:** accuracy, performance, artificial intelligence, AI, ChatGPT, large language model, LLM, difficulty index, basic medical science examination, cross-sectional study, medical education, datasets, assessment, medical science, tool, Google

## Abstract

**Background:**

Artificial intelligence (AI) has become widely applied across many fields, including medical education. Content validation and its answers are based on training datasets and the optimization of each model. The accuracy of large language model (LLMs) in basic medical examinations and factors related to their accuracy have also been explored.

**Objective:**

We evaluated factors associated with the accuracy of LLMs (GPT-3.5, GPT-4, Google Bard, and Microsoft Bing) in answering multiple-choice questions from basic medical science examinations.

**Methods:**

We used questions that were closely aligned with the content and topic distribution of Thailand’s Step 1 National Medical Licensing Examination. Variables such as the difficulty index, discrimination index, and question characteristics were collected. These questions were then simultaneously input into ChatGPT (with GPT-3.5 and GPT-4), Microsoft Bing, and Google Bard, and their responses were recorded. The accuracy of these LLMs and the associated factors were analyzed using multivariable logistic regression. This analysis aimed to assess the effect of various factors on model accuracy, with results reported as odds ratios (ORs).

**Results:**

The study revealed that GPT-4 was the top-performing model, with an overall accuracy of 89.07% (95% CI 84.76%‐92.41%), significantly outperforming the others (*P*<.001). Microsoft Bing followed with an accuracy of 83.69% (95% CI 78.85%‐87.80%), GPT-3.5 at 67.02% (95% CI 61.20%‐72.48%), and Google Bard at 63.83% (95% CI 57.92%‐69.44%). The multivariable logistic regression analysis showed a correlation between question difficulty and model performance, with GPT-4 demonstrating the strongest association. Interestingly, no significant correlation was found between model accuracy and question length, negative wording, clinical scenarios, or the discrimination index for most models, except for Google Bard, which showed varying correlations.

**Conclusions:**

The GPT-4 and Microsoft Bing models demonstrated equal and superior accuracy compared to GPT-3.5 and Google Bard in the domain of basic medical science. The accuracy of these models was significantly influenced by the item’s difficulty index, indicating that the LLMs are more accurate when answering easier questions. This suggests that the more accurate models, such as GPT-4 and Bing, can be valuable tools for understanding and learning basic medical science concepts.

## Introduction

Advances in artificial intelligence (AI), machine learning, and large language models (LLMs) have made these tools widely used across a variety of industries. Education and other fields are increasingly using these technologies for decision-making and predictive analysis, using machine learning fed by large databases [1]. Their utility has expanded to a wide range of applications, including speech recognition, image categorization, and language translation [2].

The application of computer technologies to study and create models for decision-making, prediction, and simulation is known as machine learning. Model performance is based on training datasets. The incorporation of AI into traditional health care and medical education has had a substantial impact on medical practices [3]. It has accelerated diagnostic processes in radiography [4], pathology, endoscopy, and ultrasonography, has improved clinical decision-making, and has decreased the workloads of health care personnel. AI has had an impact on pharmaceutical development and management and medical education, resulting in a new paradigm [5].

A study on the accuracy of ChatGPT in answering questions that were contextually similar to those in the United States Medical Licensing Examination (USMLE) reported accuracy rates of 44%‐64% for step 1 and 42%‐57.8% for step 2, depending on the dataset [6]. This research indicated that the model’s accuracy in answering questions matched the passing score for third-year medical students, suggesting that further development is required for ChatGPT to meet or exceed the USMLE passing criteria [7]. Additionally, the model has the potential to generate insightful content that could aid human learners in studying medical sciences [8].

Evaluations of ChatGPT’s accuracy in answering university-level physiology examination questions have shown it can correctly answer more than 75% of them. Furthermore, it can provide explanations that align with expert assessments [9]. For specialized surgical studies, ChatGPT’s GPT-4 model, an evolution of GPT-3.5, has been used to assess surgical question accuracy, revealing an overall accuracy of 76.4%, compared to 46.8% with GPT-3.5, a statistically significant difference (*P*<.05). GPT-4 showed an accuracy range of 63.6%‐88.3% across different topics, outperforming GPT-3.5 in every subtopic [10].

In terms of answering questions for family medicine experts in Taiwan, ChatGPT demonstrated an accuracy of 41.6% in a study that also found that the length of the questions did not affect the model’s accuracy. However, the authors noted that the AI’s accuracy might depend on the difficulty of the test, the local language, and medical practices, which differ by region and could reduce the model’s accuracy [11].

This study investigated the accuracy of responses from widely used LLM AIs, including ChatGPT (with GPT-3.5 and GPT-4), Bing, and Google Bard. Also, we compared their accuracy and determined relationships with the difficulty index for multiple-choice questions closely related to the content of the Thailand Center for Medical Competency Assessment step 1, as well as other factors that may affect the AI’s accuracy, such as the length of the question, the presence of negatively worded questions, and the variety of topics across various systems. This research was undertaken to explore these dimensions.

## Methods

### Study Design and Setting

This study was carried out at the Institute of Medicine, Suranaree University of Technology, Thailand. The curriculum has been accredited by the World Federation for Medical Education since 2021, and the program enrolls 92 medical students annually. Preclinical medical students receive instruction through a collaboration between the School of Preclinic, the Institute of Science, and the Institute of Medicine.

### Ethical Considerations

The Human Research Ethics Committee at Suranaree University of Technology approved an exemption (certificate of exemption 117/2566) for this study, which was conducted in accordance with international guidelines for human research.

### Data Source

**Figure 1.**
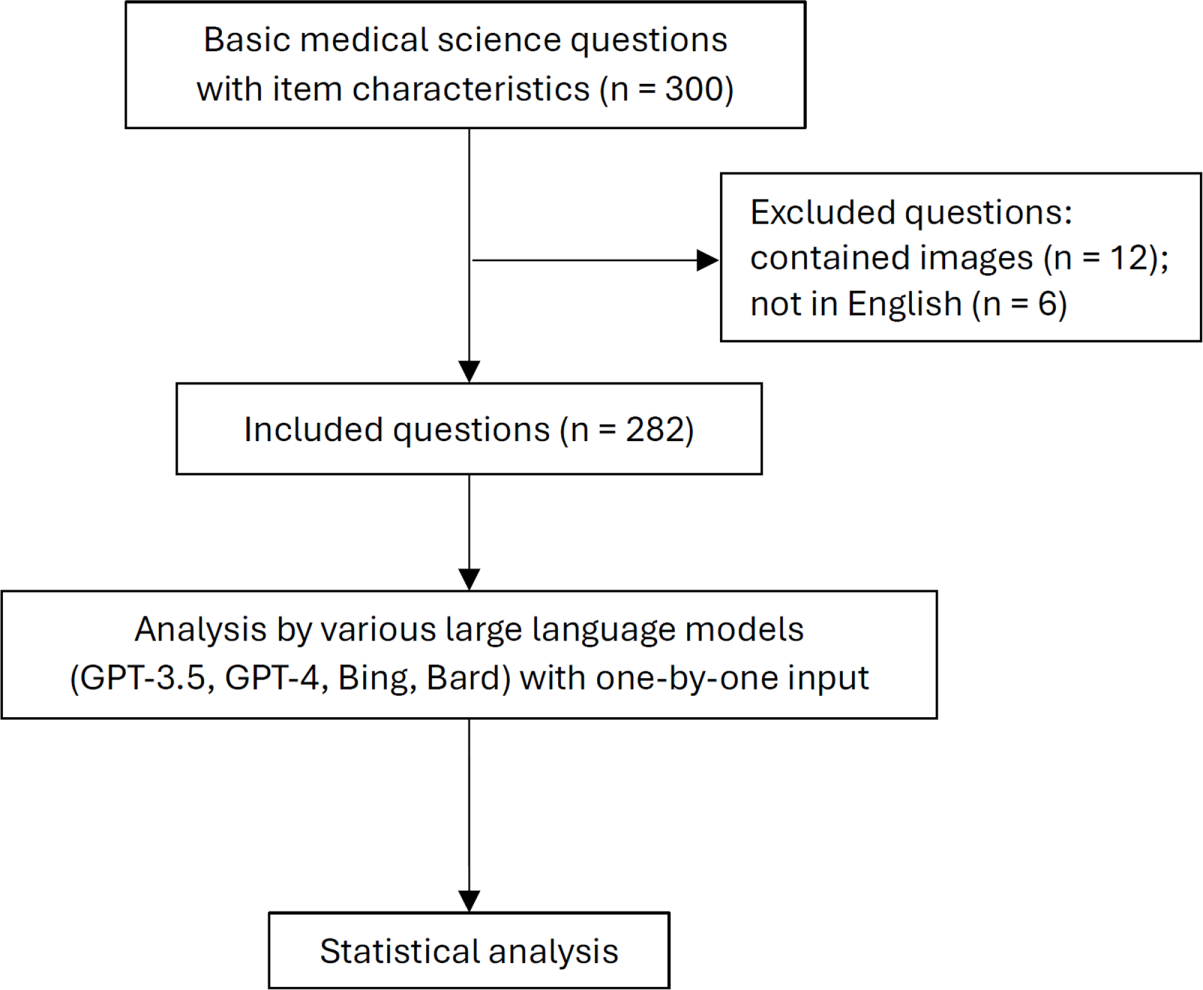
Study flow chart.

This study used a set of 300 multiple-choice questions that closely matched the content and topic distribution of Thailand’s step 1 National Medical Licensing Examination. These questions were voluntarily administered to third-year medical students in February 2021 and 2022. This timing was chosen because the students had already completed courses relevant to the examination. The difficulty index and discrimination index of each question were assessed from the test. The same set of questions was used for both years without any modifications to the content of the exam. The study excluded questions that contained pictures or were not written in English. These exclusion criteria were applied to ensure consistency in the type of questions assessed and to maintain a focus on the textual comprehension and response accuracy of the LLMs (Figure 1).

### Question Characteristics

Question length was defined as the number of words contained within a question. Negative word questions were identified as those containing the terms “not,” “no,” “exclude,” or “neither.” Case scenario questions were characterized by the inclusion of a clinical case scenario, providing a contextual background to the question being asked.

We also used item analyses [12-14], such as the difficulty index, discrimination index, and internal consistency reliability, as independent factors associated with the LLMs’ accuracy.

Difficulty index (represented by the letter *p*) is the proportion of examinees who answered a specific question correctly. If a question is easy and every examinee answers it correctly, *p* will be 1. Conversely, if no examinees answer the question correctly, *p* will be 0. This index helps in evaluating the relative difficulty of each question in an examination [12].

Discrimination index (represented by the letter *r*) refers to a question’s ability to differentiate between examinees who have high scores and those who do not. Questions with a high discrimination ability are characterized by high scorers typically answering them correctly, while low scorers tend to answer them incorrectly [13]. The most widely used metric for assessing a question’s discrimination ability is the point-biserial correlation. The point-biserial correlation coefficient ranges from −1 to 1. A higher point-biserial correlation indicates a question with better discriminatory power.

Internal consistency reliability was measured with Cronbach α. It ranges from 0 to 1, with higher values indicating greater internal consistency. A Cronbach α value above 0.7 is generally considered acceptable, values above 0.8 are considered good, and values above 0.9 are considered excellent.

### Prompt Input for LLMs

We used the prompt “Choose the best one answer.” Each question was asked to each LLM after inputting the prompt during the same period, from January 18 to 24, 2024. We individually inputted the selected questions into various LLMs, including ChatGPT (with GPT-3.5 and GPT-4), Microsoft Bing, and Google Bard (one session contained one prompt and individual question). The responses from these models were then categorized as either correct or incorrect.

### Statistical Analysis

In this study, discrete variables are represented as percentages, while continuous variables are represented as either the mean (SD) or median (IQR). The association between categorical variables was analyzed using the *χ*^2^ test or Fisher exact test. The relationships between variables and the ability of the LLMs to provide correct answers was examined using multivariable logistic regression, with results reported as odds ratios (ORs) and 95% CIs. Statistical significance was determined at a *P* value of <.05 for all tests. The analysis was facilitated by Stata (version 17; StataCorp), which was used for data analysis and chart creation.

## Results

We evaluated the LLMs by using a set of 300 multiple-choice questions that were closely aligned with the content and topic distribution of Thailand’s Step 1 National Medical Licensing Examination. According to the exclusion criteria, 12 picture-containing questions and 6 non-English questions were excluded; therefore, 282 eligible questions were included. All eligible questions were concurrently input into various LLMs (Figure 1). The responses were then recorded, categorizing the outcomes as either correct or incorrect.

The questions were categorized according to the block system (Table 1), with distributions as follows: 32.3% on general principles, 5.7% on the hematopoietic system, 8.2% on the nervous system, 3.9% on skin and connective tissues, 4.3% on the musculoskeletal system, 7.8% on the respiratory system, 8.9% on the cardiovascular system, 7.5% on the gastrointestinal system, 6.7% on the urinary system, 7.1% on the reproductive system, and 7.8% on the endocrine system. The average question length was 49.10 (SD 18.94) words, with 24 questions (8.2%) containing negative wording. More than half of the questions, specifically 53.2%, were based on clinical case scenarios (more descriptive statistics for the item analysis for each block are provided in Multimedia Appendix 1). The mean difficulty index was 0.35, indicating moderately difficult to difficult questions. The discrimination index was 0.16, suggesting a poor ability to distinguish between higher and lower performers. Otherwise, the internal consistency reliability, at 0.84, highlighted an acceptable level of consistency across the examination.

**Table 1.** Question characteristics (n=282).

Characteristics	Values
**Number of questions by block, n (%)**	
General principles^a^	91 (32.3)
Hematopoietic system	16 (5.7)
Nervous system	23 (8.2)
Skin and connective tissue	11 (3.9)
Musculoskeletal system	12 (4.3)
Respiratory system	22 (7.8)
Cardiovascular system	25 (8.9)
Gastrointestinal system	21 (7.5)
Urinary system	19 (6.7)
Reproductive system	20 (7.1)
Endocrine system	22 (7.8)
Question length (words), mean (SD)	49.10 (18.94)
Negative-word questions, n (%)	24 (8.5)
Case scenario questions, n (%)	150 (53.2)
Average difficulty index (*p*)	0.35
Average discrimination index (*r*)	0.16
Internal consistency reliability (α)	0.84

a “General principle” questions refer to fundamental principles in biochemistry, molecular biology, human development, genetics, normal immune responses, basic pathological processes, laboratory investigations, general pharmacology, epidemiology, and biostatistics.

The overall accuracy of the LLMs in the basic medical science examination was as follows (Table 2): GPT-4 achieved the highest accuracy at 89.07% (95% CI 84.76%‐92.41%), Microsoft Bing had an accuracy of 83.69% (95% CI 78.85%‐87.80%), GPT-3.5 recorded an accuracy of 67.02% (95% CI 61.20%‐72.48%), and Google Bard demonstrated an accuracy of 63.83% (95% CI 57.92%‐69.44%). The Fisher exact test showed that GPT-4 performed more accurately than Microsoft Bing, and that the difference was statistically significant (*P*<.001)

**Table 2.** Accuracy of large language models with 95% CIs, compared based on category (n=282).

	GPT-3.5	GPT-4	Microsoft Bing	Google Bard
Number of correct answers	189	251	236	180
Overall accuracy, % (95% CI)	67.02 (61.20‐72.48)	89.07 (84.76‐92.41)	83.69 (78.85‐87.80)	63.83 (57.92‐69.44)
General principles, % (95% CI)	84.62 (75.54‐91.33)	90.11 (82.05‐95.38)	84.62 (75.54‐91.33)	72.53 (62.17‐81.37)
Block system, % (95% CI)	61.78 (54.49‐68.70)	88.48 (83.08‐92.64)	83.25 (77.18‐88.25)	59.69 (52.36‐66.70)

**Figure 2.**
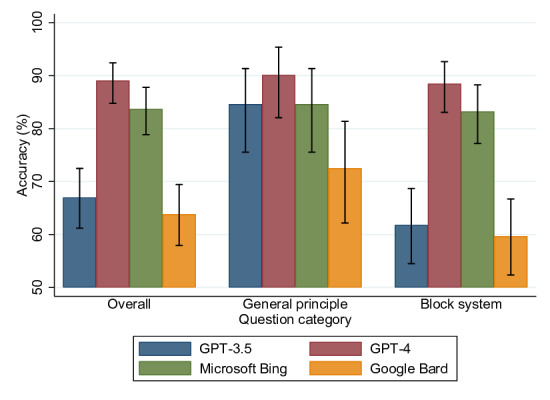
Comparative accuracy with 95% CIs for artificial intelligence models across different question categories.

The GPT-4 model demonstrated the highest accuracy among the LLMs in the general principles section for basic science, achieving 90.11% (95% CI 82.05%‐95.38%), as shown in Table 2. GPT-3.5 and Bing exhibited equal accuracy in this section, with the lowest accuracy being 72.53% (95% CI 62.17%‐81.37%) for Bard. Additionally, GPT-4 maintained its position as the top performer in the block system with an accuracy of 88.48% (95% CI 83.08%‐92.64%), whereas Bard again displayed the lowest performance in this segment (Figure 2). Overall, GPT-4 stood out for its superior performance in overall accuracy, general principles, and the block system.

Table 3 presents the number of correct answers stratified by the block system alongside the proportion of correct answers relative to the total number of questions. The GPT-4 model exhibited the best performance, with its accuracy ranging from 84% to 95%. Following GPT-4, the Microsoft Bing model demonstrated block system accuracies between 68% and 91%. The accuracy of GPT-3.5 and Google Bard was comparable in this study, with GPT-3.5 achieving between 53% and 85%, and Google Bard ranging from 53% to 72%.

**Table 3.** Number of correct answers stratified by block system (n=282)

Topic	Correct answers, n (%)			
	GPT-3.5	GPT-4	Microsoft Bing	Google Bard
General principles (n=91)	77 (85)	82 (90)	77 (85)	66 (73)
Hematopoietic system (n=16)	8 (50)	14 (88)	13 (81)	11 (69)
Nervous system (n=23)	13 (57)	21 (91)	19 (83)	12 (52)
Skin and connective tissue (n=11)	8 (73)	10 (91)	9 (82)	5 (46)
Musculoskeletal system (n=12)	9 (75)	11 (92)	10 (83)	8 (67)
Respiratory system (n=22)	12 (55)	17 (77)	19 (86)	13 (59)
Cardiovascular system (n=25)	14 (56)	21 (84)	20 (80)	16 (64)
Gastrointestinal system (n=21)	15 (71)	20 (95)	18 (86)	14 (67)
Urinary system (n=19)	10 (53)	17 (90)	13 (68)	10 (53)
Reproductive system (n=20)	13 (65)	18 (90)	18 (90)	12 (60)
Endocrine system (n=22)	16 (73)	20 (91)	20 (91)	13 (59)

Table 4 illustrates the question characteristics associated with correct answers. There was a correlation between the difficulty index and the accuracy in all 4 models, with the strongest association observed in the GPT-4 model (OR 90.13, 95% CI 4.30‐1887.54; *P*=.004). This was followed by GPT-3.5, which had an OR of 28.03 (95% CI 4.68‐167.98; *P*<.001). Microsoft Bing and Google Bard demonstrated similar correlations with correct answers, with ORs of 18.9 (95% CI 1.84‐195.42; *P*=.01) and 18.73 (95% CI 3.12‐112.45; *P*=.001), respectively, as shown in Table 4. There was no statistically significant correlation between the accuracy of GPT-3.5, GPT-4, and Bing and question length, negative word questions, clinical case scenario questions, or the discrimination index.

**Table 4.** Multivariable logistic regression analysis showing question characteristics associated with correct answer of large language model artificial intelligence (n=282).

Variable	GPT-3.5		GPT-4		Microsoft Bing		Google Bard	
	OR^a^ (95% CI)	*P* value	OR (95% CI)	*P* value	OR (95% CI)	*P* value	OR (95% CI)	*P* value
Question length (word)	0.99 (0.97‐1.00)	.07	1.00 (0.98‐1.02)	.96	1.00 (0.98‐1.02)	.94	0.98 (0.97‐1.00)	.02
Negative word question	0.55 (0.22‐1.35)	.19	0.44 (0.15‐1.30)	.14	0.46 (0.18‐1.22)	.12	0.26 (0.10‐0.69)	.007
Case scenario question	0.94 (0.50‐1.77)	.85	1.57 (0.63‐3.93)	.34	0.94 (0.43‐2.04)	.87	0.56 (0.30‐1.07)	.08
Difficulty index (*p*)	28.03 (4.68‐167.98)	<.001	90.13 (4.30‐1887.54)	.004	18.9 (1.84‐195.42)	.01	18.73 (3.12‐112.45)	.001
Discrimination index (*r*)	2.80 (0.34‐23.32)	.34	4.85 (0.20‐116.54)	.33	9.66 (0.67‐140.06)	.10	9.31 (1.02‐84.68)	.048

a OR: odds ratio.

On the other hand, for Google Bard, longer questions had a higher OR, of 0.98 (95% CI 0.97‐1.00; *P*=.02), for the model to provide the correct answer than shorter questions. The negative-word questions were less likely to be answered correctly by the model, with an OR of 0.26 (95% CI 0.10‐0.69; *P*=.007), compared to those without negative words. Furthermore, questions with a higher discrimination index were more likely to be correctly answered with statistical significance by the model, with an OR of 9.31 (95% CI 1.02‐84.68, *P*=.048), as compared to those with a lower discrimination index. No statistically significant correlation was observed between the accuracy of the AIs in answering clinical case scenario questions, as presented in Table 4.

**Figure 3.**
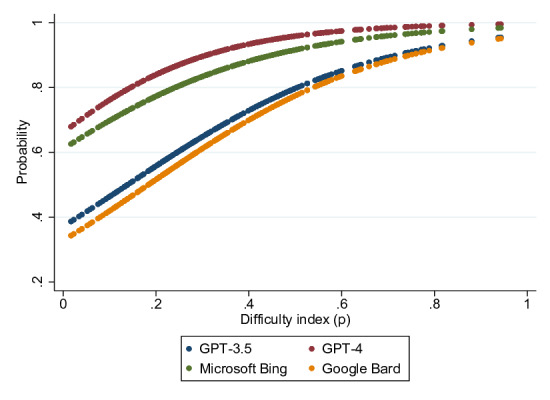
Accuracy of various artificial intelligence models estimated based on difficulty index.

The correlation between the difficulty index and the estimated accuracy of the various AI models, analyzed with binary logistic regression, is shown in Figure 3. The GPT-4 model consistently demonstrated the highest accuracy across all levels of question difficulty index (Figure 3). Google Bard, on the other hand, had the lowest estimated accuracy. The accuracy of the various LLMs improved as the difficulty index increased, indicating that these models performed better on easier questions.

## Discussion

### Accuracy of the LLMs on Basic Medical Science Examinations

This study compared the accuracy of LLMs in answering questions from a basic medical science examination related to the National Medical Licensing Examination, finding that GPT-4 had the highest accuracy, at 89.07%, and Google Bard had the lowest accuracy, at 63.83%, when tasked with answering questions in this context. The most frequently studied AI models were GPT-3.5 and GPT-4.

These results align with the 2023 findings of Yanagita et al [15], who used questions from the National Medical Licensing Examination in Japan, administered by the Japanese Ministry of Health, Labour and Welfare. When inputting Japanese questions into the prompt, they reported an accuracy for GPT-4 of 81.5%, significantly higher than GPT-3.5’s accuracy of 42.8%, with GPT-4 surpassing the National Medical Licensing Examination passing standard of 72%.

Our results are similar to those of the study conducted by Gilson et al [6] in 2023, which found that the performance of GPT-3.5 on AMBOSS-Step1 and NBME-Free-Step1 was 44% and 64.4%, respectively. Flores-Cohaila et al [16] conducted a study on the accuracy of LLMs on the Peruvian National Licensing Medical Examination and discovered that GPT-4 had 86% accuracy, following by GPT-3.5 at 77%, with moderately difficult to difficult questions being associated with incorrect answers (the OR for GPT-3.5 was 6.6, 95% CI 2.73‐15.95; for GPT-4, the OR was 33.23, 95% CI 4.3‐257.12).

A literature review from China (Wang et al [17]) evaluated the performance of GPT-3.5 and GPT-4 on the China National Medical Licensing Examination and reported 56% and 84% accuracy for GPT-3.5 and GPT-4, respectively, demonstrating GPT-4’s superiority over GPT-3.5 in terms of accuracy on basic medical science examinations.

The accuracy of GPT-4 and GPT-3.5 is influenced by the variety within the question dataset. This results in diverse outcomes across different countries, changing according to the environmental context, difficulty level of the examination, and the proportion of subcomponents within the examination question sets, which may vary from one country to another. Consequently, the estimated accuracy of AI models for each dataset is not constant.

### Difficulty Index and the LLMs’ Accuracy

In this study, we identified factors correlated with the accuracy of AI models in answering questions. We found that for every model, the difficulty index was associated with correctly answering questions. Moreover, across all models, there was a tendency to answer questions correctly as the difficulty index increased (indicating easier questions). Specifically, GPT-4 demonstrated the highest OR at 90.13 (95% CI 4.30‐1887.54; *P*=.004), followed by GPT-3.5, with an OR of 28.03 (95% CI 4.68‐167.98; *P*<.001).

This result aligns with findings from Antaki et al [18] showing that question difficulty was the most predictive factor of GPT-3.5’s answer accuracy (likelihood ratio 24.05; *P*<.001) and that GPT-4 was more accurate than GPT-3.5. The current research reveals the accuracy of AI models in answering questions across various disciplines, particularly studies focusing on the renowned GPT-3.5 model. However, this study focused on the relationship between the difficulty index, derived from human examination observations, and the accuracy of every simple-to-access LLM that is widely used. There was also a variation in accuracy among all models, with GPT-4 being the most accurate, and there was an obvious correlation with the difficulty index for each model, indicating that easier questions had higher accuracy.

### The Implication of LLMs for Medical Education

This study’s findings hold significant implications for medical education, particularly regarding the use of LLMs such as GPT-4, Microsoft Bing, GPT-3.5, and Google Bard as educational tools [19]. There are 3 major ways that this study’s findings can be applied to augment traditional study methods.

First, enhancing study efficiency: the high accuracy rates of LLMs, especially GPT-4, in answering medical examination questions suggest their utility as effective study aids. By providing immediate and accurate answers with explanations, these models can help students identify areas of weakness and reinforce their learning more efficiently than traditional study methods alone.

Second, supplementing traditional education methods: LLMs can act as supplementary tools in medical education, alongside lectures, textbooks, and clinical scenarios. Integrating LLMs into the curriculum provides students with an additional resource for study and review to enhance the overall educational experience.

Last, preparing for licensing examinations: given the study’s focus on medical licensing examinations, LLMs could play a crucial role in preparing students for these critical assessments. The ability of LLMs to accurately answer examination questions, such as those tackled by GPT-4, and explain reasoning processes can assist students in better preparing for the format and content of licensing exams.

LLMs may have a negative impact on medical education. Excessive dependence on LLMs might impede the development of independent critical thinking skills. Students may become reliant on the model’s suggestions instead of developing their own reasoning processes. LLMs can sometimes provide incorrect, incomplete, or biased information [20,21]. This can interfere with the development of critical appraisal skills, leading students to accept inaccurate information, which may hinder their critical thinking and medical reasoning abilities [22]. Additionally, reduced peer and mentor interaction can hinder the development of professional judgment, depriving students of diverse perspectives and collaborative problem-solving experiences.

To maximize the benefits while minimizing the negative impact of incorporating LLMs into medical education [23], 4 strategies can be considered. First, structured use: LLMs can be incorporated as supplementary tools in a structured curriculum rather than as primary sources of information. Second, critical appraisal training: the importance of critically appraising information provided by LLMs should be emphasized, and students should be taught how to cross-reference and validate information. Third, independent thought should be encouraged: environments should be fostered that encourage independent thinking and problem-solving, using LLMs to support (not replace) these processes. Fourth, monitoring and evaluation: the impact of LLMs on students’ learning and reasoning skills should be assessed, and educational approaches should be adjusted based on these assessments.

### Limitations

One significant limitation of this study is the LLMs’ ability to accurately respond to complex medical examination questions. Moreover, despite GPT-4’s high performance, the study’s focus on a single culturally and geographically specific medical licensing examination (Thailand Step 1 National Medical Licensing Examination) may limit the generalizability of the findings to other medical examinations and educational contexts. The exclusion of questions containing images and those not in English restricted the comprehensiveness of the assessment, considering the importance of questions on visual diagnostics. Updates to LLMs can significantly affect their accuracy, leading to a potential increase in the capabilities of the models over time. Furthermore, different LLMs can respond differently to different prompts. They can generate different answers across independent sessions, even with identical prompts. Therefore, a sensitivity analysis of the accuracy of the LLMs’ responses should be conducted with a variety of prompt and session settings.

### Conclusion

Our results show a significant variation in performance among different LLMs, with the most accurate model being GPT-4. This study has shed light on the role of LLMs as supplementary tools in medical education, as well as the need for more research to increase the generalizability of the findings to different educational settings. We advocate for the ongoing development and modification of LLMs to match the unique demands of medical education internationally, which has important implications for the future integration of AI in medical training and test preparation.

## Supplementary material

10.2196/58898 Multimedia Appendix 1 Descriptive statistics of item analysis for each block system.

## Abbreviations

AI: artificial intelligence
LLM: large language model
USMLE: United States Medical Licensing Examination
