# Supplementary material for: Factors Associated With the Accuracy of Large Language Models in Basic Medical Science Examinations: Cross-Sectional Study
Source: JMIR Med Educ. 2025 Jan 13;11:e58898. doi: 10.2196/58898 (PMC11745146; doi:10.2196/58898)
Supplement: Multimedia Appendix 1 [file mededu-v11-e58898-s001.docx]

**Supplementary material**

-----------------------------------------------------------------

**Table**: Descriptive statistic of item analysis for each block system

| **Block** | **Number** | **Question length** | **Negative word question** | **Case scenario question** | **Difficulty index** | **Discrimination index** |
| --- | --- | --- | --- | --- | --- | --- |
| General principle | 9 | 44.0 ± 17.9 | 9 (9.9) | 24 (26.4) | 0.35 ± 0.20 | 0.13 ± 0.15 |
| Hematopoietic system | 16 | 52.9 ± 18.7 | 1 (6.3) | 11 (68.8) | 0.32 ± 0.18 | 0.26 ± 0.12 |
| Nervous system | 23 | 54.8 ± 19.9 | 1 (4.4) | 18 (78.3) | 0.26 ± 0.15 | 0.10 ± 0.12 |
| Skin and connective tissue | 11 | 50.6 ± 26.5 | 1 (9.1) | 5 (45.5) | 0.28 ± 0.13 | 0.09 ± 0.14 |
| Musculoskeletal system | 12 | 50.8 ± 15.1 | 1 (8.3) | 11 (91.7) | 0.29 ± 0.09 | 0.13 ± 0.11 |
| Respiratory system | 22 | 50.0 ± 12.3 | 2 (9.1) | 13 (59.1) | 0.29 ± 0.19 | 0.13 ± 0.13 |
| Cardiovascular system | 25 | 60.7 ± 17.9 | 1 (4.0) | 21 (84.0) | 0.35 ± 0.17 | 0.21 ± 0.11 |
| Gastrointestinal system | 21 | 49.8 ± 18.2 | 1 (4.8) | 16 (76.2) | 0.40 ± 0.23 | 0.19 ± 0.16 |
| Urinary system | 19 | 51.3 ± 22.3 | 3 (15.8) | 6 (31.6) | 0.38 ± 0.26 | 0.13 ± 0.16 |
| Reproductive system | 20 | 45.3 ± 20.1 | 2 (10.0) | 12 (60.0) | 0.43 ± 0.23 | 0.18 ± 0.17 |
| Endocrine system | 22 | 47.3 ± 18.5 | 2 (9.1) | 13 (59.1) | 0.48 ± 0.23 | 0.24 ± 0.17 |

Discrete variables were represented in number and percentages, while continuous variables were presented mean ± standard deviation. General principle questions refer to fundamental principles in biochemistry, molecular biology, human development, genetics, normal immune responses, basic pathological processes, laboratory investigations, general pharmacology, epidemiology, and biostatistics.
